# A Survey of Tick-Borne Bacterial Pathogens in Florida

**DOI:** 10.3390/insects10090297

**Published:** 2019-09-13

**Authors:** Carrie E. De Jesus, Claudia Ganser, William H. Kessler, Zoe S. White, Chanakya R. Bhosale, Gregory E. Glass, Samantha M. Wisely

**Affiliations:** 1 Department of Wildlife Ecology and Conservation, University of Florida, Gainesville, FL 32611, USA; carriedejesus@ufl.edu (C.E.D.J.); zseganish@ufl.edu (Z.S.W.); 2 Department of Geography, University of Florida, Gainesville, FL 32611, USA; gancla@ufl.edu (C.G.); wkessler@ufl.edu (W.H.K.); gglass@epi.ufl.edu (G.E.G.); 3 Department of Entomology and Nematology, University of Florida, Gainesville, FL 32611, USA; cbhosale@ufl.edu

**Keywords:** tick-borne disease, Florida, surveillance, *Rickettsia*, *Borrelia*, *Ehrlichia*, *Amblyomma americanum*

## Abstract

Within the past three decades, new bacterial etiological agents of tick-borne disease have been discovered in the southeastern U.S., and the number of reported tick-borne pathogen infections has increased. In Florida, few systematic studies have been conducted to determine the presence of tick-borne bacterial pathogens. This investigation examined the distribution and presence of tick-borne bacterial pathogens in Florida. Ticks were collected by flagging at 41 field sites, spanning the climatic regions of mainland Florida. DNA was extracted individually from 1608 ticks and screened for *Anaplasma*, *Borrelia*, *Ehrlichia* and *Rickettsia* using conventional PCR and primers that amplified multiple species for each genus. PCR positive samples were Sanger sequenced. Four species of ticks were collected: *Amblyomma americanum*, *Amblyomma maculatum*, *Dermacentor variabilis,* and *Ixodes scapularis*. Within these ticks, six bacterial species were identified: *Borrelia burgdorferi*, *Borrelia lonestari*, *Ehrlichia ewingii*, *Rickettsia* *amblyommatis*, *Rickettsia andeanae*, *Rickettsia* *parkeri*, and *Rickettsia* endosymbionts. Pathogenic *Borrelia*, *Ehrlichia*, and *Rickettsia* species were all detected in the North and North-Central Florida counties; however, we found only moderate concordance between the distribution of ticks infected with pathogenic bacteria and human cases of tick-borne diseases in Florida. Given the diversity and numerous bacterial species detected in ticks in Florida, further investigations should be conducted to identify regional hotspots of tick-borne pathogens.

## 1. Introduction

The number of reported tick-borne pathogen infections has more than doubled in the United States over the past decade according to the U.S. Center of Disease Control and Prevention (CDC) [1]. In response, state and federal surveillance efforts have increased, and the majority of those efforts have been concentrated in the Northeastern and Upper Midwest of the U.S. to understand epidemiology and disease ecology of *Borrelia burgdorferi*, the causative agent of Lyme disease. However, in the Southeastern U.S. the number of tick-borne bacterial infections causing ehrlichiosis, spotted fever rickettsiosis and Lyme disease is increasing [2,3]. Surveillance in this region has detected multiple etiological agents (*Borrelia, Rickettsia*, *Ehrlichia*, and *Anaplasma*) that cause human and animal disease [2,3,4,5,6,7]. Within the past three decades, a diverse array of new bacterial etiological agents of tick-borne disease have been discovered in the U.S. including *E. chaffensis*, *E. ewingii*, *R. parkeri*, and Panola Mountain Ehrlichia sp. (PME) [8,9,10,11]. Given the discovery and diversity of tick-borne bacterial pathogens detected in the U.S., there is a substantial need for ongoing pathogen surveillance of ticks nationally.

To address the need for pathogen surveillance, the CDC has developed standardized protocols to survey for tick species and pathogens [12] in order to monitor changes in the distribution and abundance of ticks, as well as to detect the presence and estimate the prevalence of tick-borne pathogens. These data can be used as part of a larger program that addresses the questions of when and where people may be exposed to ticks and their associated pathogens [12,13]. The specific objective of the surveillance protocol is to determine the presence of ticks and their associated pathogens at the appropriate spatial scale throughout the United States [12].

**Figure 1:**
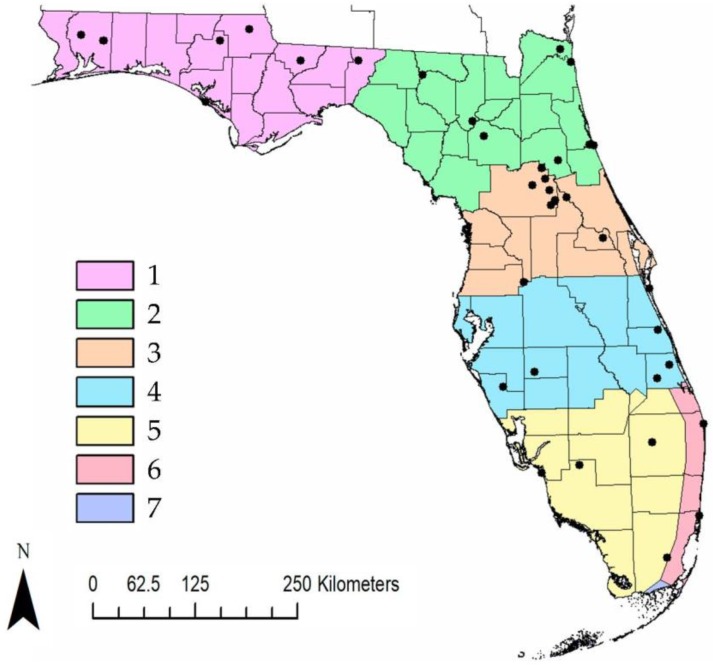
Map of the seven climatic regions of Florida. Each color represents a climatic region going from north to south. Black dots indicate field sites where ticks were collected.

Florida has been a largely neglected state in the surveillance of tick-borne bacterial pathogens. Florida is divided into seven distinct climatic regions that vary from north (temperate) to south (subtropical), making it more climatically diverse than other southeastern states (Figure 1) [14]. The variation between temperate to subtropical climate could influence the distribution of ticks and bacterial pathogens present [14,15]. Previous studies of ticks and bacterial pathogens have been restricted geographically from the Panhandle to Central Florida (regions 1–3), which are climatically similar to other southeastern states [6,13,15,16,17,18]. Florida has multiple tick species that are vectors for bacterial pathogens: *Amblyomma americanum*, *A. maculatum, Dermacentor variabilis*, *Ixodes scapularis* and *Rhipicephalus sanguineus* [15,16,17,18].

Within the last eight years, human cases of tick-borne disease have been reported in 42 of the 67 counties in Florida with >50 cases per year acquired in state (Table 1) [19]. The primary tick-borne illnesses of concern in Florida are Lyme disease, ehrlichiosis and spotted fever rickettsiosis. Cases of tick-borne diseases in Florida occur between the months of April through August. Lyme disease is the most commonly reported tick-borne disease; however, the majority of reported cases are reported to be acquired out of state. Previous studies in Florida have detected *B. burgdorferi* sensu stricto in *I. scapularis* adults and detected *Borrelia lonestari*, a bacterium of unknown pathogenicity, in *A. americanum* [20,21].

Ehrlichiosis is a locally prevalent disease in Florida. Multiple vertebrate species (white-tailed deer (*Odocoileus virginianus*), raccoons (*Procyon lotor*), wild pigs (*Sus scrofa*) and opossums (*Didelphis virginiana*) have been found to be seropositive for *Ehrlichia* species in Florida [8,18,22,23,24]. Previous studies have reported infected *A. americanum* throughout the central part of the state for both *Ehrlichia ewingii* and *E. chaffeensis* [17,18].

In Florida approximately a dozen cases of spotted fever rickettsiosis are reported each year; however, ticks are commonly infected with a variety of *Rickettsia* species that can be either endosymbionts or pathogenic to humans and animals. *Rickettsia* species that cause spotted fever rickettsiosis can induce mild to severe symptoms. *Rickettsia rickettsii*, the pathogen of Rocky Mountain spotted fever (RMSF), is the most severe of the Rickettsioses, which can result in fatality if untreated. This pathogen is mainly vectored by *D. variabilis*¸ but *A. americanum* and *R. sanguineus* are also considered vector species [25,26,27,28]. Infections of *R. rickettsii* by *R. sanguineus* have been identified in communities with peridomestic dogs heavily infested with ticks, including in Florida [27,28]. In contrast *R. parkeri* is less severe and can result in mild fever and eschar [11]. *Rickettsia parkeri* is primarily vectored by *A. maculatum* but has also been found in *A. americanum* [28]. *Rickettsia amblyommatis* has been reported as a possibly pathogenic Rickettsia that produces milder symptoms than *R. parkeri* or *R. rickettsii* and is the most common bacterial pathogen reported in *A. americanum* [4,6,28,29]. Nationwide, the number of spotted fever cases has increased, yet this trend is accompanied by a decrease in case fatality, suggesting that an increase in the prevalence of less pathogenic species like *R. amblyommatis* is the cause of the trend in human cases [30].

Florida has multiple tick species that are capable of vectoring a variety of tick-borne bacterial pathogens. Given the limited geographic scope of previous tick-borne pathogen studies yet broad geographic scope of human cases, further investigation is needed to better understand the distribution of tick-borne bacterial pathogens across the entire state. The purpose of our investigation was to examine the distribution of tick-borne bacterial pathogens within the six climatic regions of the Florida mainland. Our survey followed the guidelines of the CDC protocol as implemented by Glass et al. [31] for the detection of pathogens among distinct climatic regions. Our study discusses results in the context of the presence of pathogens and human acquired cases of tick-borne diseases to provide a more comprehensive picture of tick-associated pathogens and human exposure [13].

## 2. Materials and Methods

### 2.1. Tick Collections

We collected ticks at field sites across the 6 climatic regions of mainland Florida using the methodology of Glass et al. [31] and Kessler et al., 2018 [32]. Briefly, sampling effort was stratified across the range of climatic regions that varied across the latitudinal gradient of Florida, included 41 field sites, and incorporated 28 of the 67 Florida counties following CDC guidelines for collecting tick pathogen data (Figure 1) [12]. We collected ticks by flagging (1 m^2^, felt) on transects of 100–200 m 4–6 times a year. Ticks collected from 2016 through August 2017 were stored in 70% ethanol. Collection protocols changed in September 2017, and all samples through 2018 were stored in 100% ethanol and kept cool until they could be stored at −80 °C, where they were stored until processing. All ticks were identified by sex, life stage, and species using taxonomic keys [33,34,35,36]. Seasonality was also examined for each tick species collected.

### 2.2. DNA Extractions

We extracted only DNA from ticks collected from 2016 through August 2017. To extract DNA, adult and nymphal ticks were cut in half on a piece of parafilm using flame-sterilized forceps and scalpel. Half of each tick was then placed into proteinase K digestion and ATL buffer and digested overnight at 56 °C (QIAGEN, Valencia, CA, USA). When single larvae were found, they were directly placed into the proteinase K digestion. Once digested, the Qiagen DNeasy extraction kit (QIAGEN, Valencia, CA, USA) was used according to the manufacturer’s protocol [37]. The quality and quantity for each tick measured on a Nanodrop (Nanodrop One, Thermo Fisher Scientific, Carlsbad, CA, USA). Quality of DNA was also visualized on a gel in order to check for high molecular weight. In order to prevent contamination, DNA and RNA extractions were conducted in a separate room from the PCR preparation and products. The extracted DNA was stored at −20 °C and RNA at −80 °C until PCR protocols were implemented.

From September 2017 through October 2018, ticks were extracted using the Trizol reagent (Thermo Fisher Scientific, Carlsbad, CA, USA) in order to extract both RNA and DNA. Adult ticks were cut in half on a piece of parafilm before being placed into the Trizol buffer using a flame-sterilized and RNase-free scalpel. Nymphal ticks were cut on the bottom quarter of the tick, and singleton larvae were directly placed into Trizol. Once in Trizol, all ticks were sonicated. Adults were sonicated with 3 pulses but nymphs and larvae were processed with 2 pulses, each pulse was 5 s each at 25% amplitude (Q125 QSonica Sonicator, Newtown, CT, USA). The rest of the Trizol protocol was followed according to manufacturer instructions [38]. Once DNA and RNA were extracted, DNA was stored at −20 °C and RNA at −80 °C until PCR protocols were run.

Because we changed DNA extraction protocols, we compared both methods to determine if there were any differences in quality and quantity of the DNA using unpaired *t*-tests. In addition, we conducted a logit regression to determine if the quantity of DNA had an impact on our ability to amplify bacterial DNA. All statistical analyses were conducted using the base package in R [39].

### 2.3. Pathogen Screening

Our pathogen screening methods allowed for the detection of a wide array of tick-borne bacterial pathogens of concern to human health by using primers that detected multiple species within a genus of bacteria. Ticks were screened for *Anaplasma*, *Ehrlichia, Borrelia*, and *Rickettsia* species. For *Ehrlichia* and *Anaplasma* the PCR targeted the groESL gene, *Borrelia* targeted the Flagellin b gene, and *Rickettsia* the ompA gene (Table 2) [40,41,42]. Individual adults, nymphs, and larvae were tested for each bacterial pathogen.

PCR amplification protocols were conducted on a Master cycler pro S (Eppendorf, Hamburg, Germany). Positive controls used in the PCR protocols were derived from DNA extractions from an *A. americanum* that was positive for *E. chaffeensis* and *B. lonestari*. For the *Rickettsia* positive control, DNA from a *Rickettsia* spp. endosymbiont was extracted from an *I. scapularis*. Lastly, a supernatant of infected dog cells was extracted for the positive control of *A*. *phagocytophilum*. A negative control was also run for each PCR assay with PCR grade water. PCR products were run on a gel using RedView DNA Gel Stain™ (Genecopoeia, Rockville, MD, USA) to determine if the gene sequences of interest were amplified. Samples were considered positive for a bacterium if a band for the appropriate sequence size was present on a gel. All samples that tested positive for a pathogen were sent to GenScript (Piscataway, NJ, USA) for Sanger sequencing. Sequences were aligned using Geneious (v 11.1) and then compared to sequences in GenBank using NCBI BLAST. All pathogen sequences matched sequences available in the NCBI database. Genetic sequences for each bacterial species were submitted to NCBI.

## 3. Results

### 3.1. Tick Collections

In total 1608 ticks were collected from March 2016 through October 2018 (Table 3). Ticks were collected from all six climatic regions and 20/28 counties surveyed by flagging. Of the ticks collected, *A. americanum* (*n* = 1325) was the most abundant and was found in 16/28 counties (Table 3). In addition, 233 *I. scapularis* were collected in 14/28 counties and 46 *D. variabilis* in 4/28 counties (Table 3). Lastly, *A. maculatum* was the least abundant tick, with only two nymphs and two adults collected in 4/28 counties. *Amblyomma* and *Dermacentor* ticks were abundant during the spring and summer months, while *Ixodes* adults were more abundant during the winter months.

### 3.2. Pathogen Screening

**Figure 2:**
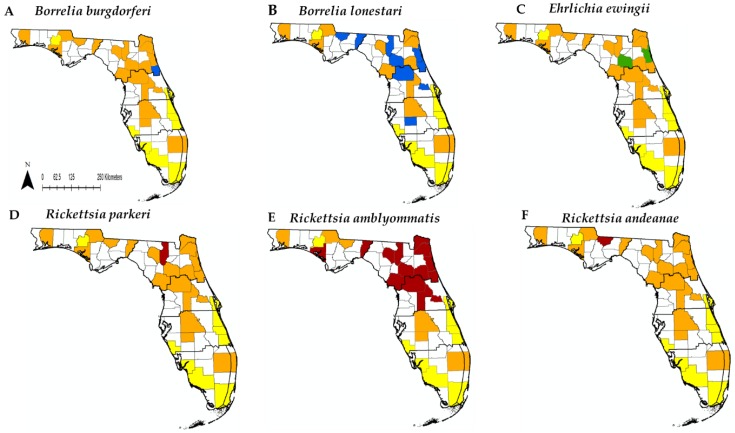
Florida counties where ticks were surveyed, and ticks that were positive for a bacterial species. Yellow = site surveyed but ticks not collected, Orange = counties where ticks were collected but were not infected with a bacterial species. Red/blue/green = county with positive ticks for a genus of bacterial species detected. *Ixodes scapularis* vectors: Thick black lines denote climatic regions. (**A**) *B. burgdorferi*. *Amblyomma americanum* vectors: (**B**) *B. lonestari*, (**C**) *E. ewingii*, (**D**) *R. parkeri*, (**E**) *R. amblyommatis. Amblyomma maculatum* vectors: (**F**) *R. andeanae*.

**Figure 3:**
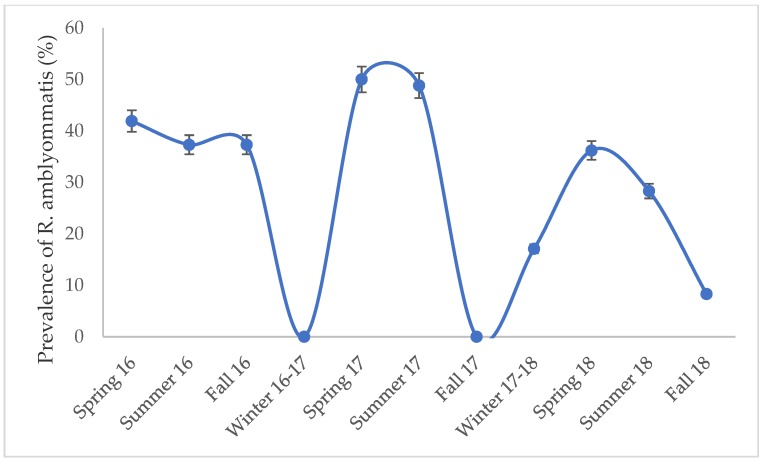
Prevalence ±95% confidence interval (CI) of *R. amblyommatis* in all life stages of *A. americanum* over a three-year collection period. *Amblyomma americanum* were not collected in winter 1–17 and fall 17.

We found ticks that tested positive for *Borrelia*, *Ehrlichia*, and *Rickettsia* species, but no *Anaplasma* species were detected (Figure 2, Table 4). Two species of *Borrelia* were detected; *B. burgdorferi* and *B. lonestari*. *B. burgdorferi* was detected in one adult *I. scapularis* and *B. lonestari* in 16 *A. americanum* and one *D. variabilis*. For *Ehrlichia* only one species was detected, *E. ewingii*. Two *A. americanum* tested positive for *E. ewingii*. The two ticks infected with *E. ewingii* were also co-infected with *R. amblyommatis*. *R. amblyommatis* was the most abundant bacteria found in ticks with 410 infected *A. americanum* detected. *R. amblyommatis* had the highest prevalence compared to all other pathogen species detected and was most prevalent in ticks collected from the spring and summer months (Figure 3). Two *A. americanum* larvae were positive for *R. parkeri*. *R. andeanae* was detected in 1 *A. maculatum*. Lastly, *I. scapularis* and *D. variabilis* were both found to be infected with *Rickettsia* endosymbionts.

### 3.3. Comparison of DNA Extraction Protocols

DNA concentrations used in PCR ranged from 1.91–158.4 ng for Qiagen and 2.5–420.8 ng for Trizol protocols. For the Qiagen protocol the average DNA concentration for adults, nymphs and larvae were 37.87 (95% CI, ±2.82), 18.22 (±2.14), and 20.05 ng/µl (±14.74). The Trizol protocol averages for DNA concentrations were 92.15 (±4.83), 130.36 (±6.72), and 199.80 ng/µL (±73.93). The Trizol extracted significantly more DNA compared to the Qiagen protocol (*t*-test, *t* = 34.4, df = 1412.3, *p* < 0.0001). We had an average 280/260 value of 1.67 (±0.22) for the Trizol protocol and 1.79 (±0.02) for Qiagen. We did see significant difference between the 280/260 (*t* = 3.317, df = 1103.2, *p* = 0.001). For the 260/230 values we obtained an average value of 1.34 (±0.19) for the Trizol and 1.31 (±0.18) for the Qiagen protocol. We did not see significant differences in the 260/230 values between both extraction protocols (*t* = 1.3545, df = 1637.9, *p*-value = 0.1758). DNA quantity of DNA did not impact our PCR results for the Trizol (logit, df = 1041, z = −0.073, *p* = 0.941) and Qiagen protocols (logit, df = 686, *z* = 0.153, *p* = 0.878).

### 3.4. Pathogen Detection and Human Cases in Florida

**Figure 4:**
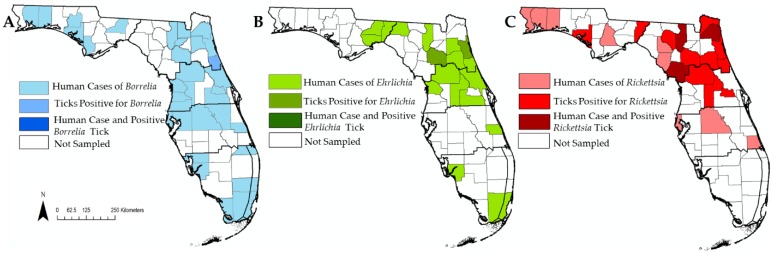
Maps of counties with reported human cases, tick detected with a pathogen and overlap between human cases and ticks infected with a pathogen. Thick black lines denote climatic regions. (**A**) Map of human cases of Lyme disease and detection of ticks with *B. burgdorferi*. (**B**) Map of human cases of Ehrlichiosis and detections of ticks infected with *E. ewingii*. (**C**) Map of human cases of Ehrlichiosis and detections of ticks infected with pathogenic *Rickettsia* species.

*Borrelia*, *Ehrlichia* and *Rickettsia* were all detected within climatic regions 1–3. (Figure 3). *Borrelia burgdorferi* was only detected in one county within climatic region 2 (Figure 3) even though human cases for locally acquired *B.burgdorferi* infection were spread throughout the entire state (Figure 4). *Borrelia lonestari* was detected across climatic regions 1–3 and the only pathogen detected in region 4. *Ehrlichia ewingii* was found in climatic region 2. Our pathogen survelliance for *Ehrlichia* did not overlap with human cases at the county level. Human cases of Ehrlichiosis did overlap with climatic regions 2–3 and with a few cases in regions 4–6. Human cases of rickettsiosis were concentrated in climatic regions 1–3, with a few cases in region 4. *Rickettsia*-postive ticks were found throughout cimatic regions 1–3. Ticks infected with *Rickettsia* overlapped with reported human cases for spotted fever rickettsiosis at the county level within climatic region 2 (Figure 4). Ticks were collected in climatic region 4 but they were not infected with *Rickettsia*, even though human cases of rickettsiosis have been reported in the region. 

## 4. Discussion

The purpose of our investigation was to examine the distribution of tick-borne bacterial pathogens across the climatic regions of mainland Florida. We found four tick species and six bacterial species in 4/6 climatic regions. All the tick and pathogen species we detected had previously been reported in Florida but our survey provided information on distribution of ticks and bacterial pathogens within a larger geographic context [6,15,16,17,43].

In our survey, *Amblyomma americanum* was the most abundant tick sampled in Florida from which multiple bacterial pathogens were detected, which is consistent with tick surveys across the Southeastern U.S. [43]. *Rickettsia amblyommatis* was the most common bacterial species detected in our study and is a highly prevalent bacteria across the Southeastern U.S. [3,4,6,29]. *Rickettsia amblyommatis* has been reported as a potentially pathogenic *Rickettsia* with mild symptoms [29,30]. In the U.S. there has been a decrease in hospitalizations and fatalities for RMSF cases but an increase in mild cases [30]. This trend is thought be associated with spread of *A. americanum* vectoring *R. amblyommatis* and *A. maculatum* vectoring *R. parkeri*, which both cause milder illness than RMSF. Further complicating the epidemiology, the causative agent of spotted fever rickettsiosis in the U.S. is either not identified or diagnostic tests used to test for RMSF cross react with other spotted fever *Rickettsia* species [44,45]]. Thus, the clinical and epidemiological picture of rickettsiosis remains unclear. Florida has roughly a dozen reported cases of spotted fever rickettsiosis each year, but diagnostic testing is rarely performed, which causes variation year by year if cases are reported as confirmed or probable [19]. The true infectivity of *R. amblyommatis* and *R. parkeri* could be underreported in the state given the mild symptoms of and imprecise diagnostic tools for these infections.

*Amblyomma maculatum* is considered the primary vector of *R. parkeri* [46]; however, our survey did not detect *R. parkeri* in this tick but instead we detected *R. andeanae*. *R. andeanae* has been identified as a spotted fever *Rickettsia*, but its pathogenicity is still unknown [47]. Populations of *A. maculatum* infected with *R. andeanae* vary across the Midwestern and Southeastern U.S. In Oklahoma and Kansas, *A. maculatum* infected with *R. andeanae* had a prevalence of 47–80% [48], but in southeastern states, including Florida, prevalence of *R. andeanae* is <5% [48]. Previous investigations have found that hard ticks cannot maintain dual infections of *Rickettsia* by vertical transmission, which is referred to as rickettsial interference [49]. It is plausible, given that *A. maculatum* can vector *R. parkeri* and *R. andeanae*, that rickettsial interference maybe occurring and influencing the distribution of these *Rickettsia* species across Florida.

*Dermacentor variabilis* is an important vector for RMSF, which we did not detect in our study. Previous investigations have found that *R. rickettsii* is rare in *D. variabilis* in the U.S. [50]. We did detect *B. lonestari* and *Rickettsia* endosymbionts in our *D. variabilis* samples. *Borrelia* species have been previously detected in *D. variabilis* such as “*Candidatus Borrelia texasensis*” [51,52], which has only been detected in Webb County, Texas [51,52]. *Dermacentor variabilis* has also been shown to have bacterial species found in *A. americanum* including *E. chaffeensis* and *E. ewingii,* although we did not detect these pathogens in *D. variabilis* in this study [52].

*Ixodes scapularis* is a highly competent vector for multiple bacterial pathogens, and its role as a disease vector is poorly understood in Florida. In our investigation we only detected one *I. scapularis* with *B. burgdorferi*; however, our survey methods of *I. scapularis* relied solely on flagging methods and likely underestimated the distribution of low-prevalence pathogens like *Borrelia* in Florida. Differences in behavior between *I. scapularis* in the north and the southeast may have influenced our sample sizes and the ability to detect the bacteria. Because *I. scapularis* in the Northeastern U.S. quest above or on top of leaf litter and *I. scapularis* in the Southeastern U.S. quest under the leaf litter, flagging may not efficiently capture this tick species in Florida [53]. Nonetheless, flagging mimics human activity and is a good indicator of human encounters with different tick species and the pathogens to which they may be exposed [54].

In our survey we found that the temperate climatic regions (regions 1–3), which are similar in climate to the rest of the Southeastern United States [14], also had a similar tick-borne pathogen community to other southeastern regions [8,21,22]. In the subtropical regions of Southern Florida (climate regions 4–6), we found fewer ticks and fewer infected ticks compared to northern climate regions in Florida. Subtropical environments typically have higher amounts of precipitation during the summer months, which can influence the presence and survival of ticks [32]. This finding could also be a product of sampling bias. Dragging instead of flagging could be more appropriate for collecting ticks in subtropical parts of Florida because it may sample ticks that are more active in the leaf litter in hotter, more humid climates [53,54]. Previous studies have successfully collected ticks in Southern Florida with dragging and by surveying mammalian hosts, including deer and wild pigs [15,16,55]. Human cases of tick-borne pathogens are also present in the subtropical regions of Florida, suggesting that humans and ticks are coming in to contact even if they are not detected by flagging.

In addition to our tick and pathogen surveillance, we wanted to qualitatively assess if the distribution of infected ticks collected by flagging was similar to human case data. Visually comparing maps of both datasets, we did not find substantial overlap at the county scale with human cases and ticks infected with pathogens (Figure 4). However, our datasets only compared data collected in 2016–2017; in previous years human cases of Ehrlichiosis had been reported in counties where we found ticks to be positive for pathogens [19]. Even though we did not see direct pathogen and human case overlap, comparing both epidemiological and acarological data can still provide valuable insight by providing different perspectives on disease risk [13]. Epidemiological data alone can skew where cases are located, since the location of human case does not always correspond to where an infection was acquired and can inflate area of potential risk. However, epidemiological data do provide evidence that humans are encountering etiological disease agents. Acarological data provide information on vector distribution, abundance and pathogen prevalence. Previous investigations combining both types of data have been used to develop risk maps for Lyme disease in California, which found that areas with high tick abundance were associated with areas with high human case incidence at the subcounty level [13].

Future studies of tick pathogen surveillance in Florida should continue to incorporate the use of epidemiological and acarological data but consider differences in scale of either surveillance effort [13]. In our investigation we looked at bacterial pathogens at the scale of climatic regions. We did find overlap between infected ticks and human cases at this larger spatial scale; however, we did not see overlap of human cases and bacterial pathogens at the small county-level scale. In the California Lyme disease risk study, they found that habitats differed at the sub-county level, which influenced tick distribution [13]. Further tick bacterial pathogen surveillance in Florida should consider finer scale surveillance to account for differences in habitat types.

Lastly, we examined the seasonal patterns of ticks and *R. amblyommatis*. We found that *D. variabilis* and *A. americanum* were most active during the spring and summer months, while adult *I. scapularis* were active during the winter. Since, *Rickettsia amblyommatis* was the most abundant pathogen detected that we examined seasonality, which peaked during the spring and summer months (Figure 3). Epidemiological data for tick-borne diseases report that most human cases occur throughout the spring and summer months, which corresponds with our pathogen survey data (Table 3 and Table 4) [19]. Future pathogen prevalence studies should aim to collect ticks during spring and summer months when ticks and pathogens are active and pose the greatest risk to human health.

In our investigation we were not able to address the prevalence of tick-borne bacterial pathogens. Our sampling methodology focused on collecting ticks from numerous field sites in order to provide a state-wide overview of pathogen presence based on CDC guidelines, but not estimates of prevalence. Previous studies in Florida have reported low prevalence (0–5%) of numerous tick bacterial pathogens [6,7,17,18,20]. In order to estimate the prevalence of these pathogens in future studies in large tick populations with a confidence level of 0.95, a sample size of ~300 ticks per site would be needed to accurately estimate a pathogen with the true prevalence of 1% [56].

## 5. Conclusions

Our study has provided an initial baseline for tick pathogen distribution for the state of Florida. We found multiple medically important tick species and tick-borne bacterial pathogens. Ticks and pathogens were concentrated within climatic regions 1–3 and were limited in distribution in the southern regions of the state [19]. We did not find overlap between human cases and pathogen surveillance at the county level but did at the climatic region level. Future surveillance efforts should account for scale in order to account for habitat variation within a county. Given the diversity of ticks and tick-borne bacterial pathogens in Florida, further investigations should be conducted to identify regional hotspots of tick-borne pathogens in order to fully determine the risk to public health.

## Figures and Tables

**Table 1:** Acquired cases of tick-borne diseases in Florida. Includes confirmed and probable cases (Florida Department of Health, Morbidity and Mortality Report 2011–2017).

Disease	2011	2012	2013	2014	2015	2016	2017
**Anaplasmosis**	6	1	0	0	0	1	0
**Ehrlichiosis**	13	20	17	23	16	23	12
**Lyme disease**	22	39	21	35	35	36	27
**Spotted fever rickettsiosis**	9	20	15	21	12	7	15

**Table 2:** Published PCR assays used in this survey. The table includes the targeted gene, primer sequence and positive controls.

Pathogen	Target Gene	Primer Sequences 5’-3’	Positive Control	Reference
*Anaplasma/Ehrlichia*	groESL	**gro607F** GAAGATGC(A/T)GT(A/T)GG(A/T)TGTAC(G/T)GC **gro677F** ATTACTCAGAGTGCTTCTCA(A/G)TG **gro1294R** AG(A/C)GCTTC(A/T)CCTTC(A/T)AC(A/G)TC(C/T)TC **gro1121R** TGCATACC(A/G)TCAGT(C/T)TTTTCAAC	*E. chaffensis*	[40]
*Borrelia*	Flagellin b	**FlaLL** ACATATTCAGATGCAAGACAGA **FlaRL** GCAATCATAGCCATTGCAGATTGT **FlaLS** AACAGCTGAAGAGCTTGGAATG **FlaRS** CTTTGATCACTTATCATTCTAATAGC	*B. lonestari*	[41]
*Rickettsia*	ompA	**Rr 190.70p** ATGGCGAATATTTCTCCAAAA **190.701** GTTCCGTTAATGGCAGCATCT	*R.sp* endosymbiont	[42]

**Table 3:** Species identity and sample size of ticks screened across the entire state of Florida between spring 2016 and fall 2018.

Species	Spring 16	Summer 16	Fall 16	Winter 16–17	Spring 17	Summer 17	Fall 17	Winter 17–18	Spring 18	Summer 18	Fall 18	Total
***A. americanum***												
Adult	60	45	0	0	52	0	5	34	33	173	0	402
Nymph	133	89	0	8	75	0	75	30	238	106	156	910
Larvae	0	2	0	2	0	0	1	6	2	0	0	13
***A. maculatum***												
Adult	0	0	0	0	0	0	1	0	0	1	0	2
Nymph	0	0	0	0	1	0	0	1	0	0	0	2
***D. variabilis***												
Adult	6	8	0	0	5	0	2	1	10	8	6	46
***I. scapularis***												
Adult	10	1	0	94	62	0	7	18	36	2	0	230
Nymph	2	0	0	1	0	0	0	0	0	0	0	3

**Table 4:** Pathogen prevalence of ticks collected from Spring 2016 through Fall 2018. * Most commonly reported BLAST result for each pathogen.

Tick Species	Pathogen Species	No. Positive/No. Tested (%) [95% CI]	% Identical (Accession #) *
*I. scapularis*	*B. burgdorferi*	1/233 (0.43%) [0–0.02]	98% (AF264895.1)
*I. scapularis*	*R. endosymbiont*	80/233 (34.3%) [0.3–0.42]	98% (AB002268)
*D. variabilis*	*R. endosymbiont*	1/46 (2.2%) [0–0.11]	97% (AB002268)
*D. variabilis*	*B. lonestari*	1/46(2.2%) [0–0.11]	99% (AF273670)
*A. americanum*	*B. lonestari*	17/1312 (1.29%) [0.01–0.02]	100% (AF273670)
*A. americanum*	*E. ewingii*	2/1312 (0.16%) [0–0.001]	98% (AF195273)
*A. americanum*	*R. amblyommatis*	391/1312 (29%) [0.27–0.32]	100% (CP003334)
*A. americanum*	*R. parkeri*	2/1312 (0.16%) [0–0.01]	99.7% (MH247927)
*A. maculatum*	*R. andeanae*	1/4 (25%) [0.05–0.7]	100% (KY628370)
